# Beyond Chiral Organic (p-Block) Chromophores for Circularly Polarized Luminescence: The Success of d-Block and f-Block Chiral Complexes

**DOI:** 10.3389/fchem.2020.00555

**Published:** 2020-07-28

**Authors:** Benjamin Doistau, Juan-Ramón Jiménez, Claude Piguet

**Affiliations:** Department of Inorganic and Analytical Chemistry, University of Geneva, Geneva, Switzerland

**Keywords:** circularly polarized luminescence (CPL), coordination complexes, lanthanides, chromium(III), dissymmetry factor

## Abstract

Chiral molecules are essential for the development of advanced technological applications in spintronic and photonic. The best systems should produce large circularly polarized luminescence (CPL) as estimated by their dissymmetry factor (*g*_lum_), which can reach the maximum values of −2 ≤ *g*_lum_ ≤ 2 when either pure right- or left-handed polarized light is emitted after standard excitation. For matching this requirement, theoretical considerations indicate that optical transitions with large magnetic and weak electric transition dipole moments represent the holy grail of CPL. Because of their detrimental strong and allowed electric dipole transitions, popular chiral emissive organic molecules display generally moderate dissymmetry factors (10^−5^ ≤ *g*_lum_ ≤ 10^−3^). However, recent efforts in this field show that *g*_lum_ can be significantly enhanced when the chiral organic activators are part of chiral supramolecular assemblies or of liquid crystalline materials. At the other extreme, chiral Eu^III^- and Sm^III^-based complexes, which possess intra-shell parity-forbidden electric but allowed magnetic dipole transitions, have yielded the largest dissymmetry factor reported so far with *g*_lum_ ~ 1.38. Consequently, 4f-based metal complexes with strong CPL are currently the best candidates for potential technological applications. They however suffer from the need for highly pure samples and from considerable production costs. In this context, chiral earth-abundant and cheap d-block metal complexes benefit from a renewed interest according that their CPL signal can be optimized despite the larger covalency displayed by d-block cations compared with 4f-block analogs. This essay thus aims at providing a minimum overview of the theoretical aspects rationalizing circularly polarized luminescence and their exploitation for the design of chiral emissive metal complexes with strong CPL. Beyond the corroboration that f–f transitions are ideal candidates for generating large dissymmetry factors, a special attention is focused on the recent attempts to use chiral Cr^III^-based complexes that reach values of *g*_lum_ up to 0.2. This could pave the way for replacing high-cost rare earths with cheap transition metals for CPL applications.

## Introduction

Chirality has raised human interest probably because of its ubiquity in nature (Barron, 2008), which originates from stereoselective reactions affording proteins, DNA, or more generally natural compounds (Mori, 2011). Chemists have then invested major efforts and energy to reproduce the nature efficiency with the artificial preparation of a plethora of chiral molecules and structures, such as curved aromatics (Rickhaus et al., 2017; Dhbaibi et al., 2019), coordination compounds (Knof and von Zelewsky, 1999; Seeber et al., 2006; Crassous, 2009, 2012), polyoxometalates (Hasenknopf et al., 2008), topologically complex architectures (Chambron et al., 1993; Jamieson et al., 2018), self-assembled systems (Mateos-Timoneda et al., 2004; Hembury et al., 2008; Liu et al., 2015), dynamics motors, and shuttles, to name a few (Leigh and Pérez, 2006; Feringa, 2007; Kudernac et al., 2011; van Leeuwen et al., 2017). In optics, the appeal for chiral systems is justified by their ability to rotate the plane of polarized light, a phenomenon that was discovered by the French scientist Jean-Baptiste Biot in collaboration with Jean-François Persoz in 1812. Subsequently, the physicist Aimé Cotton discovered the optical rotatory dispersion in 1895 and highlighted that chiral molecules displayed different absorption coefficients for right- and left-handed circularly polarized light, the amplitudes of which depend on the light wavelengths (Cotton effect). The latter feature gave birth to circular dichroism (CD) spectroscopy (Beychok, 1968; Schellman, 1975; Woody, 1995). The CD technique was extensively used to study the conformation and the configuration of enantiopure organic compounds (Berova et al., 2007; Pescitelli et al., 2011) and permitted to analyze the chirality and helicity of sophisticated supramolecular self-assembled architectures in solution (Pescitelli et al., 2014). Similarly to CD, which is based on the selective absorption of circularly polarized photons, some emissive chiral compounds may display a difference in intensity between right- and left-handed circularly polarized emitted light upon excitation with non-polarized light (Richardson, 1979; Riehl and Richardson, 1986; Riehl and Muller, 2004; Zinna and Di Bari, 2015; Longhi et al., 2016). The first circularly polarized luminescence (CPL) emission was observed in 1948 by Samoilov for a sodium uranyl acetate crystal. In 1960, Oosterhof reported the first CPL measurement in solution for an organic compound, namely, trans-b-hydrindanone, and for a coordination complex [Cr(en)_3_]^3+^ where en stands for ethylenediamine (Emeis and Oosterhoff, 1967). Several decades later, the development of CPL was promoted by the need for chiroptical features in versatile technological fields (Wu et al., 2015; Brandt et al., 2017), such as optoelectronics (Han et al., 2018), nanomaterials (Sang et al., 2019), chiral molecular conductors (Réthoré et al., 2005), magnetochiral devices (Rikken and Raupach, 1997; Train et al., 2011), chiral molecular switches (Feringa et al., 2000; Browne and Feringa, 2011; Isla and Crassous, 2016; Isla et al., 2016), macrostructure solvers (Roose et al., 2016), unidirectional motion controllers (Huck et al., 1996), semiconductor transistors (Yang et al., 2013), and CP-OLEDs (Gilot et al., 2010; Zinna et al., 2017).

## Physical Basis of CPL and Strategies to Achieve Strong CPL Emission

The development of CPL techniques required a quantificative parameter for comparing the amount of circularly polarized light emitted by a specific chromophore, the latter factor being independent of the non-polarized photophysical features. The dissymmetry factor *g*_lum_ (−2 < *g*_lum_ <2) was thus set as the ratio between the difference of left and right circularly polarized emission and the global emitted intensity (Equation 1). Although this value is defined for each wavelength of the CPL spectrum, it is usually reported for the maximum intensity of the emission band.

(1) $$\begin{array}{r} g_{\text{lum}}=\frac{\Delta I}{12I}=\frac{I_{\text{L}}-I_{\text{R}}}{12\left(I_{\text{L}}+I_{\text{R}}\right)} \end{array}$$

The time-dependent intensity difference of emitted light Δ*I* depends on the orientation of molecules regarding the selected polarization of the emitted light. Moreover, the excitation process is affected as well by molecule orientation since the transition probability depends on the relative orientation of the molecule transition dipole moment with respect to the incident electric field (Riehl and Muller, 2004). Consequently, working with crystals, solid samples, or more generally anisotropic media requires the explicit consideration of anisotropy in theoretical models. For matters of simplicity, the following review is restricted (i) to studies performed in isotropic media (solution) and (ii) to molecules showing excited-state lifetime one order of magnitude longer than their rotational correlation time, in order to allow relaxation to randomize the orientations of the molecules after selection of some preferential orientations upon excitation (Riehl and Muller, 2004). At room temperature, this latter criterion is fulfilled for excited-state lifetimes longer than 10 ns for molecules around 1 kD (Tóth et al., 1996).

The amplitude of the CPL signal for a *i*→*j* transition is governed by its rotatory strength (*R*_ij_), which represents the probability of emitting (or absorbing for CD) a circularly polarized photon. In an isotropic medium, *R*_ij_ can be expressed by Equation (2), where ψ_i_ and ψ_j_ are the wave functions of the initial and final states, respectively. $\hat{\mu }$ and $\hat{m}$ are the electric and magnetic perturbation Hamiltonians, while $\vec{\mu_{\text{i}\text{j}}}$ and $\vec{{\text{m}}_{\text{i}\text{j}}}$ are respectively the electric transition dipole moment (ED) and magnetic transition dipole moment (MD) and θ is the angle between those two latter vectors.

(2) $$\begin{array}{r} R_{\text{ij}}=-i\langle \psi_{\text{i}}|\hat{\mu }|\psi_{\text{j}}\rangle \langle \psi_{\text{i}}|\hat{m}|\psi_{\text{j}}\rangle =-i\;\vec{\mu_{\text{i}\text{j}}}\;▪\vec{\;{\text{m}}_{\text{i}\text{j}}}=|\vec{\mu_{\text{i}\text{j}}}|\;|\vec{{\text{m}}_{\text{i}\text{j}}}|\;\cos\theta \end{array}$$

Any contribution arising from the coupling between the electric dipole (ED) and electric quadrupole (EQ) moments is neglected in the rotatory strength equation since the consideration of an isotropic medium implies the orientation average (Gendron et al., 2019). The comparison of CPL intensities of different compounds showing different quantum yields requires some normalization of the rotatory strength by the global emission contribution which is ruled by the dipole strength (*D*_ij_) depicted in Equation (3), where $\vec{{\text{Q}}_{\text{i}\text{j}}}$ is the electric transition quadrupole moment (Gendron et al., 2019).

(3) $$\begin{array}{r} D_{\text{ij}}={\left|\vec{\mu_{\text{i}\text{j}}}\right|}^{2}+\;{\left|\vec{{\text{m}}_{\text{i}\text{j}}}\right|}^{2}+\;{\left|\vec{{\text{Q}}_{\text{i}\text{j}}}\right|}^{2} \end{array}$$

The electric transition quadrupole moment being several orders of magnitude weaker than its dipole counterpart, it is often negligible and will be omitted in the following. The dissymmetry factor can now be related to the theoretical features with Equation (4).

(4) $$\begin{array}{r} g_{\text{lum}}=\frac{4R_{\text{ij}}}{D_{\text{ij}}}=4\frac{|\vec{\mu_{\text{i}\text{j}}}|\;|\vec{{\text{m}}_{\text{i}\text{j}}}|\;\cos\theta }{{\left|\vec{\mu_{\text{i}\text{j}}}\right|}^{2}+\;{\left|\vec{{\text{m}}_{\text{i}\text{j}}}\right|}^{2}} \end{array}$$

For organic chromophores involving parity-allowed ED- and MD-forbidden transitions, the contribution of the magnetic transition dipole moment is often neglected in the denominator ($\left|\vec{\mu_{\text{i}\text{j}}}|>>|\vec{{\text{m}}_{\text{i}\text{j}}}\right|$), and the dissymmetry factor reduces to $g_{\text{lum}}=4\left(\left|\vec{{\text{m}}_{\text{i}\text{j}}}|/|\vec{\mu_{\text{i}\text{j}}}\right|\right)\cos\theta$. However, this latter equation is too restrictive and cannot be applied for magnetic dipole-allowed and electric dipole-forbidden transitions, such as those found in f–f transitions, for which the amplitude of magnetic transition dipole moment can overpass its electric counterpart (Metcalf and Richardson, 1994). The introduction of a ratio $r=|\vec{\mu_{\text{i}\text{j}}}|/|\vec{{\text{m}}_{\text{i}\text{j}}}|$ into Equation (4) permits its rewriting as Equation (5), which highlights that a maximum value of *g*_lum_ is reached when the electric and magnetic transition dipole moments are (i) colinear (θ = 0 or 180°) and (ii) of the same amplitude ($\left|\vec{\mu_{\text{i}\text{j}}}|=|\vec{{\text{m}}_{\text{i}\text{j}}}\right|$; *r* = 1) (Wada et al., 2018). The latter criterion contrasts with the usual misleading statement saying that $\left|\vec{{\text{m}}_{\text{i}\text{j}}}\right|$ should be maximized while $\left|\vec{\mu_{\text{i}\text{j}}}\right|$ is minimized, a situation encountered for organic chromophores upon neglecting $\left|\vec{{\text{m}}_{\text{i}\text{j}}}\right|$ at the denominator of Equation (4). Nevertheless, the weak magnitude of magnetic dipole compared to electric dipole transition moments ($\left|\vec{\mu_{\text{i}\text{j}}}|=10^{6}|\vec{{\text{m}}_{\text{i}\text{j}}}\right|$) indicates the ED-forbidden/MD-allowed transitions are promising for equalizing both transition dipole moments.

(5) $$\begin{array}{r} g_{\text{lum}}=4\frac{r}{r^{2}+1}\cos\theta \end{array}$$

From a theoretical point of view, the organic chromophores thus appear to be not particularly promising for CPL. However, their low cost, their easy synthetic accessibility, and the chemical inertness of carbon chemistry bring substantial advantages which (i) allow chiral resolution, (ii) afford enantiomerically pure compounds, and (iii) provide easy processability for material applications. As a consequence, CPL was systematically recorded and explored for myriads of organic chromophores exploiting central, axial, planar, or helical chirality (Sánchez-Carnerero et al., 2015; Chen and Yan, 2018; Tanaka et al., 2018a) with record values for 1,8-naphthalimide (|*g*_lum_(450 nm)| = 0.014) (Sheng et al., 2016), oligothiophenes (|*g*_lum_(520 nm)| = 0.0093) (Benincori et al., 2018; Dhbaibi et al., 2019) cyclo-2,8-chrysenylene (|*g*_lum_(443 nm)| = 0.152) (Sato et al., 2017), and 1,1′-bitriphenylenes (|*g*_lum_(449 nm)| = 0.032) (Sawada et al., 2012). Planar and helical chiralities, as found in helicenes, were highlighted to promote important dissymmetry factors because of the larger magnetic transition dipole moments induced by aromatic distortion. Hence, important efforts were made to rationalize this observation with the calculation of $\left|\vec{\mu_{\text{i}\text{j}}}\right|$ and $\left|\vec{{\text{m}}_{\text{i}\text{j}}}\right|$ and the determination of the θ angle (Sato et al., 2017; Ito et al., 2018; Tanaka et al., 2018b,c). The outstanding |*g*_lum_| value of 0.152 reported for the single-wall carbon nanotubes (Dhbaibi et al., 2019) cyclo-2,8-chrysenylene by Isobe and Sato was attributed to the unusually important magnetic transition dipole moment originating from a cyclic aromatic structure which allows and facilitates a magnetic field-induced current loop (Sato et al., 2017). In addition, due to the circular shape of the molecule, the first individual singlet-state electric transition dipole moments of the four chromophoric moieties might result in some compensation effects, as stated by Anderson for achiral nanorings (Sprafke et al., 2011). Hence, the combination of peculiar MD strength and ED weakness provides a large *g*_lum_ value. Apart from this exceptional chiral single-wall carbon nanotube, the dissymmetry factors lie in the 10^−5^−10^−2^ range for most of organic compounds (Sánchez-Carnerero et al., 2015; Chen and Yan, 2018; Tanaka et al., 2018a).

Despite the chemical advantages of organic molecules for application in material sciences (easy synthetic access, low cost, easy incorporation into devices, and processability) (Yang et al., 2013; Brandt et al., 2017; Han et al., 2018; Sang et al., 2019), the need for important dissymmetry factors prompted chemists to look into d-block chemistry. In particular, the second- and third-row metal complexes have attracted important attention due to (i) the synthetic accessibility, (ii) the chemical inertness due to large ligand-field strengths, (iii) the processability, and (iv) some easy characterization by NMR techniques for common diamagnetic Ir^III^, Pt^II^, Ru^II^, or Os^II^ complexes. In addition, the important covalency in those complexes produces highly emissive charge transfer states which are not quenched by d–d states located at much higher energies. However, the associated dissymmetry factors still cover the expected range for ED-allowed/MD-forbidden transitions (10^−5^-10^−2^) (Han et al., 2018).

It is worth noting here that significant enhancement of *g*_lum_ could be observed occasionally in nano-assemblies or in aggregated systems as a result of the formation of chiral microstructural materials or films (Geng et al., 2003; Kumar et al., 2015; Roose et al., 2016; Sang et al., 2019), in particular for few electroluminescent systems, such as OLEDs (Brandt et al., 2016; Song F. et al., 2018). In addition, the incorporation of emissive moieties into anisotropic media, such as chiral liquid crystals displaying helical macrostructures, proved to be an efficient strategy for the generation of strong CPL emission (Watanabe and Akagi, 2014; Goto, 2018; Gao et al., 2019; Kim et al., 2019; Li et al., 2019; Yang X. et al., 2019). Focusing on isotropic solutions, some recent up-converted CPL studies evidenced enhanced *g*_lum_ compared to their downshifted counterparts. This observation suggests that triplet–triplet annihilation could be an innovative and unexpected approach to increase the CPL emission of organic compounds showing weak *g*_lum_ (Han et al., 2017a; Yang D. et al., 2019; Yang X. et al., 2019).

Further optimization of the dissymmetry factors requires the operation of MD-allowed/ED-forbidden transitions in complexes showing little covalence to avoid the relaxation of the parity rules due to the mixing of metal and ligand wave functions. In this context, the lanthanide f–f transitions and the first-row d–d transitions, for which covalence is still reasonably weak, appear as promising candidates to generate important *g*_lum_. The high potential of these coordination compounds led to extensive theoretical studies in order to tackle potential spectral/structure relationships (Richardson, 1979; Riehl and Richardson, 1986; Riehl and Muller, 2004). The most comprehensive model used for rationalizing the influence of stereogenic centers on the optical activity (CD or CPL) is referred to as the independent systems/perturbation model (ISP) (Mason, 1971; Richardson, 1971; Mason and Seal, 1975, 1976; Strickland and Richardson, 1976). According to this approach, the rotatory strength emerges from two distinct mechanisms: the static coupling (SC) and the dynamic coupling (DC). The rotary strength due to SC arises from the intrinsic chirality of the emissive center which is, in the case of the coordination complexes, restricted to the metal and the ligand donor atoms. Intrinsic chirality of the first coordination sphere is ensured, for instance, for a *D*_3_ symmetric transition metal or for a lanthanide complex displaying a distorted square anti-prismatic geometry with an optimal computed twist angle of 22.5° (Carr et al., 2012; Zinna and Di Bari, 2015). If the chromophore (i.e., the coordination sphere) is achiral, while a stereogenic center is located on a remote site (i.e., a chiral ligand), the optical activity is driven by the DC mechanism. In this case, the optical activity of the metal center derives from a multipole (metal)–dipole (ligand) interaction between the emissive metal's transition density and the stereogenic center's induced dipole moment. This coupling provides a non-orthogonal contribution of the electric transition dipole moment with respect to its magnetic counterpart, the scalar product of which affords non-zero terms for the rotatory strength. When the DC mechanism is solely active, the CPL intensity mainly depends on the electrostatic quadripole–dipole interaction, which is optimized for (i) highly polarizable stereogenic centers, (ii) short metal–stereogenic center distance (the quadripole–dipole interaction distance decreases as 1/*r*^8^), and (iii) energy matching between the optical transitions of the aromatic stereogenic center and those centered on the metal (Richardson, 1979; Zinna and Di Bari, 2015). Although the intrinsic chirality of the metal coordination sphere (SC) might appear to neophyte as the best tool for maximizing *g*_lum_, the DC mechanism appears to be very efficient and may provide considerable rotatory strength. To date, the record *g*_lum_ = 1.38 belongs to the Cs[Eu(hfbc)_4_] (hfbc = heptafluorobutyrylcamphorate) complex, in which an essentially achiral square anti-prismatic coordination sphere (twist angle of −41.4°) results in a weak SC contribution, whereas the location of a camphor unit on the bound ligand is responsible for the impressive dissymmetry factor via the DC mechanism (Lunkley et al., 2008, 2011; Zinna and Di Bari, 2015).

Nonetheless, harnessing the potential of lanthanide f–f or of first-row d–d transitions requires overcoming the kinetic lability of the associated complexes, which hampers enantiomeric separations and/or provides efficient racemization pathways. In a first attempt to exploit labile lanthanide complexes, a chiral ligand is simply used to induce solely a DC mechanism. Actually, this approach is at the origin of the record dissymmetry factor reported to date (Aspinall, 2002; Lunkley et al., 2008, 2011). An alternative strategy to generate enantiomerically pure systems takes advantage of chiral ligands for inducing stereoselectivity within the coordination process (Cantuel et al., 2004; Gregolinski et al., 2008; Kotova et al., 2013). In this latter case, both SC and DC mechanisms should be active. Finally, the selection of some (rare) inert coordination complexes followed by chiral resolution and separation may afford enantiomerically pure complexes. Inert lanthanides complexes can be obtained for polydentate ligands (i.e., cryptates, DOTA) (Gregolinski et al., 2008; Walton et al., 2011; Carr et al., 2012; Dai et al., 2019) or *via* the design of supramolecular assemblies, in which the lanthanide is sequestered within inert stoppers (Zare et al., 2017a). Although the first-row transition metals are known to produce labile complexes, the preparation of inert complexes is possible with Cr^III^ (d^3^) or Co^III^ (low-spin d^6^) metals which possess large ligand-field stabilization energies, and with small and highly charged Fe^III^ centers (Hilmes et al., 1977; Cantuel et al., 2004; Dee et al., 2019; Jiménez et al., 2019). When emissive states can be implemented, those latter cheap transition metal complexes should be good candidates to provide large dissymmetry factors after chiral resolution.

## The Success of Lanthanide Complexes Using Chiral Organic Ligands and (SUPRA)Molecular Control

### Theoretical Aspects of f–f Transitions

**Figure 1:**
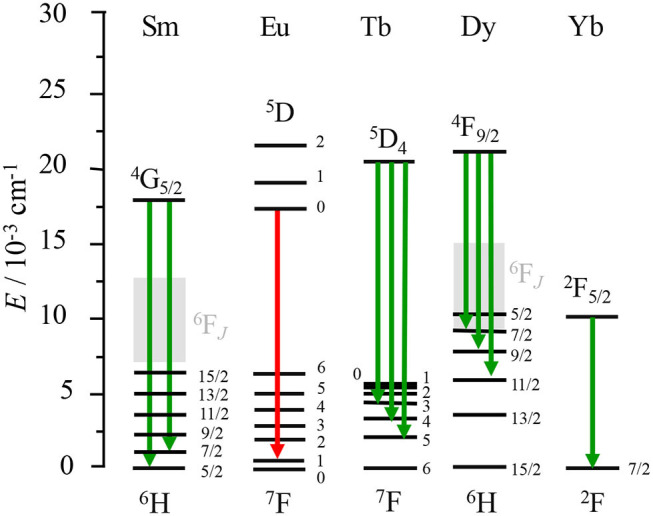
Dieke diagram of the energy of the spectroscopic levels (^2S+1^L_*J*_) for some Ln^3+^. Green arrows represent the magnetic dipole (MD)-allowed and electric dipole (ED)-allowed transitions whereas the red one represents the optimal case of an MD-allowed and ED-forbidden transition.

The absorption and emission properties of Ln^3+^-based complexes arise from the parity-forbidden and spin-forbidden metal-centered f–f transitions, the proscribed character of which can be relaxed by spin–orbit coupling and by crystal-field effects (see below). Consequently, these transitions have generally low but no negligible molar absorption coefficients (<10 M^−1^·cm^−1^) and limited radiative rate constants ($i_{\text{rad}}<10^{3}$ s^−1^). This results in long-lived excited states that give extended emissions reaching up to 10 ms for Ln^3+^ doped in low-phonon materials but reduced by one to four orders in magnitudes in Ln^3+^-based molecular complexes possessing high-energy oscillators. According to quantum mechanics, in the absence of spin–orbit coupling, any electronic wave functions can be separated into two independent contributions arising from the spin wave function and the orbital wave function. Since the interaction between an electromagnetic wave responsible for the electronic transition only involves the orbital wave function, the orthogonal and normalized character of the non-perturbated spin wave functions implies that an electronic transition between two states is only possible when the spin does not change (Δ*S* = 0), a first condition known as the spin rule. The interaction between the electromagnetic wave and the orbital part of the electronic wave functions further implies that an electric-dipole-allowed electronic transition between two states requires a parity change between the two incriminated orbital wave functions (Δ*L* = ±1, ±3…), a condition often referred to as Laporte's rule. However, magnetic dipole transitions are allowed in the absence of change in parity (Δ*L* = 0, ±2…). Accordingly, f–f transitions obeying the spin rule are electric dipole forbidden but magnetic dipole allowed. The considerable spin–orbit coupling constants which characterize the 4f-block series indeed mix orbital and spin wave functions so that the spin-selection rule (Δ*S* = 0) is relaxed for 4f−4f transitions. Concerning the parity rules controlling the electronic transitions, two different situations have to be considered. For centrosymmetric systems, the electronic f–f transitions are electric-dipole (ED) forbidden by the Laporte selection rule, but some intensity can be gained through the operation of symmetry-allowed but weak magnetic-dipole (MD, oscillator strength 10^−6^, Table 1) transitions and electric quadrupole (EQ, oscillator strength 10^−10^, Table 1) transitions. In molecular complexes, minor vibronic coupling mechanisms cannot be completely ruled out. For non-centrosymmetric systems produced by weak static crystal-field effects, the Laporte rule is partially relaxed and the mixing of electronic configurations with opposite parity gives rise to the so-called Judd–Ofelt forced ED transitions, possessing oscillator strength in the order of 10^−4^ (note that allowed ED transitions have oscillator strengths in the 0.01–1 range) (Malta and Carlos, 2003; Tanner, 2013). Altogether, the original selection rules based on spin *S* and orbital *L* quantum numbers should be replaced with some looser versions of them, which rely on the total momentum *J* quantum number (Table 1, column 5). In this context, the f–f transitions with Δ*J* = 0, ±1 (except 0↔0) are MD allowed and can benefit from the large magnetic moments displayed by these ions. Consequently, the weak ED transition mechanisms do not always dominate the transition dipole moment, as it does in organic molecules and in the major part of d-block complexes. This can lead to large rotatory strengths comparable with the dipole strength, and in the context of CPL that can afford large dissymmetry factors, *g*_lum_ (Equation 4). For instance, the ^5^${\text{D}}_{0}\to^{7}$F_1_ transition of Eu^3+^ is an example of an MD-allowed and ED-forbidden transition that leads to large *g*_lum_, typically, *g*_lum_ > 0.1 (Harada et al., 2009; Dai et al., 2016; Neil et al., 2016; Uchida et al., 2016; Zercher and Hopkins, 2016; Kono et al., 2017; Leonzio et al., 2017). Exceptionally, the complex Cs[Eu[(+)-(hfbc)_4_] displays *g*_lum_ = +1.38 at 595 nm (^5^${\text{D}}_{0}\to^{7}$F_1_), which represents the highest dissymmetric factor reported to date (Lunkley et al., 2008). The emissive ^5^${\text{D}}_{0}\to^{7}$F_2_ and ^5^${\text{D}}_{0}\to^{7}$F_3_ transitions of the aforementioned complex that do not satisfy the MD selection rule indeed yield lower but still remarkable dissymmetry factors of >0.2. Surprisingly, the Sm^3+^-based complexes possessing two MD-allowed ^4^${\text{G}}_{5/2}\to^{6}$H_5/2_ and ^4^${\text{G}}_{5/2}\to^{6}$H_7/2_ transitions have been considerably less exploited for CPL although the analog Cs[Sm[(+)-(hfbc)_4_] has been shown to produce large dissymmetry factors of +1.15 and −1.15 at 553 and 598 nm, respectively (Lunkley et al., 2011). Other chiral Sm^3+^ complexes have been also reported (Petoud et al., 2007; Kreidt et al., 2018; Cotter et al., 2019). Aside from Eu^3+^ and Sm^3+^, other lanthanides ions, such as Nd^3+^, Tb^3+^, Dy^3+^, and Yb^3+^ have been used in chiral complexes as optically active luminescence-polarized emitters, and they will be described in the following section (Maupin et al., 1998, 2000; Dickins et al., 1999; Morita et al., 2000; Petoud et al., 2007; Casanovas et al., 2017; Leonzio et al., 2017; Górecki et al., 2018; Zinna et al., 2019). Concerning the MD contribution to the total transition dipole moment in a f–f transition, Richardson classified those into types DI, DII, and DIII where DI is expected to exhibit the largest dissymmetric factor (e.g., ^5^${\text{D}}_{0}\to^{7}$F_1_ in Eu^3+^) and DII and DIII lower values, for instance, ^4^${\text{G}}_{5/2}\to^{6}$H_5/2_ and ^5^${\text{D}}_{4}\to^{7}$F_5_ in Sm^3+^ and Tb^3+^, respectively, which are type DII transitions (Richardson, 1980). With this in mind, the trivalent lanthanide Ln^3+^ ions gathered in Figure 1 represent a straightforward choice, within the periodic table, to induce strong *g*_lum_ in molecular complexes and materials. However, several key requirements must be fulfilled in order to obtain efficient (high quantum yields) luminescent chiral complexes with a strong dissymmetry factor for realistic applications.

**Table 1:** Selection rules for electronic transitions of lanthanides ions (Eliseeva and Bünzli, 2010; Tanner, 2013).

**Type of transition**	**Parity**	**|Δ*S*|**	**|Δ*L*|**	**|Δ*J*|**	**Order of magnitude of the oscillator strength**
Electric dipole (ED)	Opposite	0	≤ 1^a^	≤ 1^b^	~0.01–1
Judd forced ED	Same	0	= 0, 1, 3, 5^a^^,^ ^c^ = 2, 4, 6^a^^,^ ^d^	= 0, 1, 3, 5^b^^,^ ^c^ = 2, 4, 6^b^^,^ ^d^	~10^−4^
Magnetic dipole (MD)	Same	0	0	≤ 1^b^^,^	~10^−6^
Electric quadrupole (EQ)	Same	0	≤ 2^e^	≤ 2^f^	~10^−10^

a *L = 0 ↔ L′ = 0 are forbidden*.

b *J = 0 ↔ J′ = 0 are forbidden*.

c *If J and J′ ≠ 0*.

d *If J or J′ = 0*.

e *L = 0 ↔ L′ = 0, 1 are forbidden*.

f *J = 0 ↔ J′ = 0, 1 are forbidden*.

### Strategy for Achieving Highly Luminescent CPL Emission Using Ln^3+^

**Figure 2:**
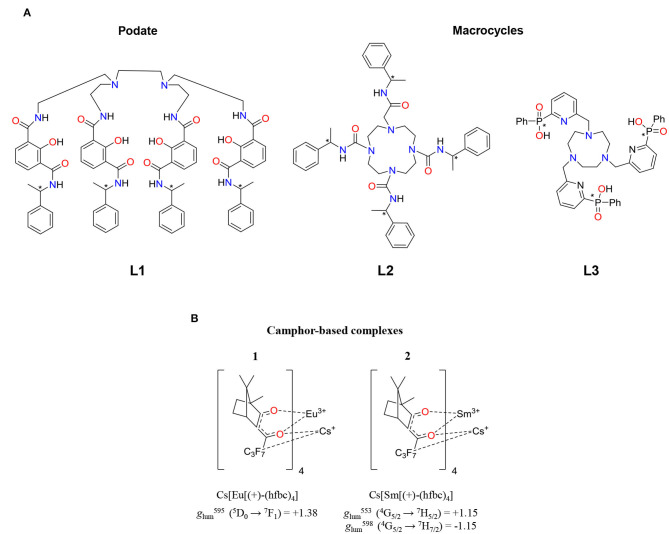
**(A)** Structure of the podate ligand (L1) reported by Raymond and coworkers for the preparation of chiral Sm, Eu, Tb, and Dy complexes. Macrocycle-based cyclen (L2) and triazacyclononane (L3) used by Parker and coworkers for the preparation of chiral lanthanide-based CPL probes (Parker et al., 2002; Petoud et al., 2007; Neil et al., 2016). **(B)** Isostructural Eu^3+^ and Sm^3+^ complexes, Cs[Ln((+)-hfbc)_4_] (Ln = Eu^3+^ or Sm^3+^), and their corresponding record *g*_lum_ values associated with their MD-allowed transitions.

A classical strategy to overcome the low molar absorptivity of f–f transitions relies on the coordination of trivalent lanthanides, Ln^3+^, to an organic chromophore, which can act as an efficient antenna, when excited, followed by an energy transfer onto the close lanthanide metal ion. This mechanism results in an efficient population of the lanthanide excited states that can relax to the ground state *via* radiative (luminescence) or non-radiative pathways. Therefore, to prepare highly luminescent chiral Ln^III^-based complexes, the following points should be kept in mind. (i) The organic chromophore should be able to harvest a considerable amount of energy by absorbing UV-Vis light and to efficiently transfer it onto the lanthanide-excited states. (ii) The ligand should favor high coordination numbers for protecting Ln^III^ from interactions with solvent molecules and subsequent non-radiative de-excitation pathways. (iii) The kinetic inertness and thermodynamic stability of the resulting complex is of major importance since dissociation could lead to the formation of several luminescent species in solution and to a decrease in the luminescence intensity. (iv) The resulting complex should preferably not be fluxional or at least it should display a slow racemization rate constant compared to the time scale of the experiment (Metcalf et al., 1990). (v) Enantiopure species are desired in order to get unambiguous structure/property relationships and to induce maximum CPL performances. On the other hand, the chirality of lanthanide complexes may arise from (i) the helical twist of the ligands around the metal ions, (ii) the stereochemical orientation of the ligating arms of multi-dentate ligands, or (iii) the incorporation of stereocenters directly onto the ligand scaffold. As regards the first two options, the coordination of an achiral ligand (e.g., DPA = dipicolinic acid or tpy = terpyridine) to a lanthanide ion indeed produces the Δ or *M* (right-handed) and Λ or *P* (left-handed) isomers in racemic proportions. However, chiral resolution with the help of chromatographic techniques are quite challenging because of the kinetically fast interconversion (racemization) occurring between the two enantiomers (Kreidt et al., 2017). Therefore, the use of enantiopure chiral ligands (point iii, above) appeared to be the most promising strategy to prepare chiral lanthanide complexes, although the chiral induction of the Ln coordination sphere is not always quantitative and the associated stereoisomeric excess is only sporadically reported. In this context, Raymond and coworkers reported on a chiral octadentate ligand (**L1**) that strongly binds Ln^3+^ and protects it from interactions with solvent molecules (Figure 2A) (Petoud et al., 2007). The authors were able to prepare a family of luminescent complexes emitting in the visible range (Sm^3+^, Eu^3+^, Tb^3+^, and Dy^3+^) with modest to reasonable quantum yields (0.8–62%) and CPL activities. Remarkable dissymmetry factors could be measured for these complexes with *g*_lum_(Eu(**L1**)) = ±0.296(2) at 596 nm, *g*_lum_(Tb(**L1**)) = ±0.046(2) at 543 nm, *g*_lum_(Sm(**L1**)) = −0.027 at 565 nm and −0.028 at 597 nm, and *g*_lum_(Dy(**L1**)) = 0.013 at 669 nm. It is worth noting that this contribution reported on the first chiral Sm^3+^ complex showing CPL activity. From a more application-oriented point of view, Parker and coworkers provided numerous examples of versatile macrocycles (e.g., cyclen and triazacyclononane) bearing different coordinating side arms decorated with N-donors, amides, phosphinates, and carboxylate groups that are able to both complex and sensitize lanthanide ions for ultimate biomolecule sensing. Chirality elements can be introduced within the ring or within the side arms of these versatile scaffolds (**L2** and **L3** in Figure 2A).

**Figure 3:**
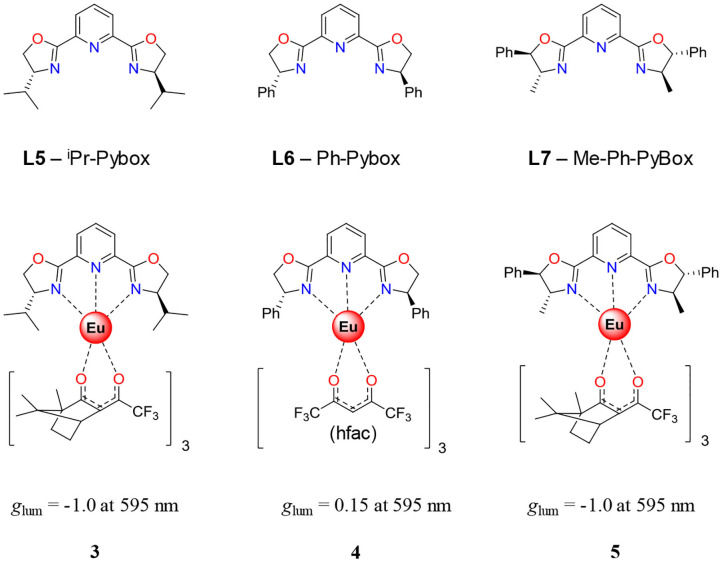
(Top) Widely used enantiomerically pure ligand (L5, L6, and L7) used for the preparation of (bottom) chiral lanthanide complexes with the corresponding *g*_lum_ associated with the Eu^III^(^5^${\text{D}}_{0}\to^{7}$F_1_) transition.

These nitrogen-containing macrocycles served as starting materials for the synthesis of a huge number of Ln^3+^ complexes with remarkable thermodynamic stabilities and kinetic inertness. The most famous is probably 1,4,7,10-tetraazacyclododecane-1,4,7,10-tetraacetic acid (DOTA) (Figure 2A, **L2**) which usually gives nine-coordinated complexes when one additional water molecule is bound to the lanthanide (Parker et al., 2002). However, lanthanide complexes based on DOTA derivatives exist as four dynamically interconvertible stereoisomers which can be best described as two enantiomeric pairs (Aime et al., 1999). This has an unfavorable effect on the CPL performance. The introduction of chiral substituents into the cyclen ring and/or into the pendant arms of the DOTA derivative drives the formation of one stereoisomer over the others (Dickins et al., 1997, 1999; Ranganathan et al., 2002; Woods et al., 2003). This is of crucial importance for controlling the degree of local helicity and the associated dissymmetry factor (Carr et al., 2012; Dai et al., 2019). These complexes have been widely used as CPL probes for sensing small molecules and proteins thanks to the great sensitivity of the CPL signals of Tb^3+^, Eu^3+^, Dy^3+^, Yb^3+^, and Nd^3+^ to conformational and structural modifications (Beeby et al., 2000). Dissymmetry factors |*g*_lum_| around 0.5 correspond to the largest values reported for Eu^3+^ and Tb^3+^ chiral DOTA-based complexes. Triazonane-based europium complexes based on **L3** (Figure 2A) and **L3** derivatives are powerful tools for anion sensing (Neil et al., 2016). It is worth noticing that **L3** in its tautomeric form (tautomery between P–OH and P=O) is achiral, but it becomes a persistent stereochemical element when coordinated to a metal. In fact, Parker's group prepared a family of highly emissive Eu complexes, the chiroptical properties of which could be induced upon addition of chiral anions. Furthermore, the same authors exploited this property for the analysis of enantiomeric purity. Other complexes with interesting chiroptical properties have been reported, and most of them belong to the family of β-diketonate ligands. These bidentate anionic ligands are efficient to coordinate Ln^3+^ because of both the oxophilicity of Ln^3+^ and the electrostatic attraction between the cationic Ln^3+^ and the anionic ligands. Furthermore, β-diketonates are good sensitizers for inducing Ln^3+^ emission since they possess strong π → π^*^ transitions usually located in the UV region. The introduction of different substituents at the 1 or 3 position allows some energy tuning of the ligand triplet state of the antenna effect but also of the crystal field splitting affecting the ^2S+1^L_*J*_ terms of the coordinated lanthanide complexes (Gálico et al., 2019). Taking advantage of the versatility of β-diketonate ligands, chiral derivatives can be easily prepared using cheap enantiopure ketones. Hence, the Eu^3+^ and Sm^3+^ complexes based on the hfbc ligand [hfbc = (+)-3-heptafluorobutylyrylcamphorato, Figure 2B] were straightforwardly prepared. They gave rise to extraordinary chiroptical properties affording the highest *g*_lum_ reported to date (+1.38 at 595 nm for the Eu^3+^ derivative). This value exceeds the previous record of −0.78 at 588 nm found for a similar camphor-based ligand, Cs[Eu((-)-facam)_4_] where facam is 3-trifluoroacetyl-d-camphorato (Riehl and Richardson, 1986; Riehl and Muller, 2004). In its pivotal work, Muller and coworkers examined the CPL properties of M^I^[Eu((+)-hfbc)_4_] complexes (M^I^ = Cs and Na) in CHCl_3_ and EtOH. They found that the magnitude of |*g*_lum_| is affected by the size of the alkali counter-ion and, to a less extend, by the nature of the solvent. In going from Cs^+^ to Na^+^, |*g*_lum_| drops to 0.15, a 9-fold reduction compared to 1.38 found for the Cs^+^ derivative. This difference has been associated with a decrease in the degree of helical twist and hence of the chiral environment of the complex, upon reducing the size of the interacting alkali cation (Lunkley et al., 2011). Last but not least, a solvent dependence was also observed in going from CHCl_3_ to EtOH, thus giving rise to lower dissymmetry factors of +1.32 and +0.06 at 595 nm for the Cs^+^ and Na^+^ derivatives. Subsequently, Di Bari and coworkers pointed out the crucial role played by the centered β-diketonane π → π^*^ transitions on the dynamic coupling mechanism (DC) operating in these complexes (Di Pietro and Di Bari, 2012). Alternatively, the combination of achiral β-diketonate antenna, including acetylacetonate (acac) or hexafluoracetylacetonate (hfac), with commercially available chiral tridentate ligands, such as 2,6-bis[(4R)-4-phenyl-2-oxazolinyl]pyridine (**L6**, Ph-Pybox) or analogous **L5** (iPr-Pybox) and **L7** (Me-Ph-Pybox) represents an efficient approach for preparing enantiopure heteroleptic chiral trivalent lanthanides with high emission properties and interesting CPL properties (Figure 3, top) (Górecki et al., 2018). For instance, by changing the nature of the ancillary ligand in going from [Eu(**L5**)(hfbc)_3_] to [Eu(**L7**)(hfbc)_3_] (Figure 3, bottom), *g*_lum_ remains similar. However, the replacement of the chiral β-diketonate co-ligand (facam) with achiral acac in [Eu(**L6**)(acac)_3_] resulted in a considerable drop in magnitude of the dissymmetry factor (Figure 3, bottom). This points out empirically the importance of having a large amount of chiral centers around the Ln^3+^ coordination environment for enhancing the dissymmetry factor. By replacing Eu^3+^ with Yb^3+^, Di Bari and coworkers obtained [Yb(**L5**)(tta)_3_] and [Yb(**L6**)(tta)_3_] (tta = thenoyltrifluoroacetone), for which |*g*_lum_| values of 0.019 and 0.029 could be recorded for the ^2^${\text{F}}_{7/2}\to^{2}$F_5/2_ transition (Zinna et al., 2019), one of the rare examples of the NIR-CPL signal reported up to date.

Axial chiral ligands, such as (*R*/*S*)-2,2′-bis(diphenylphosphoryl)-1,1′-binaphthyl (BINAPO) have been also proven to be appropriate for inducing CPL (Harada et al., 2009; Liu et al., 2018; Cotter et al., 2019). Chiral terpyridine (tpy) ligands allowed Muller and coworkers to prepare helical complexes (Ln = Eu^3+^ and Tb^3+^) which showed poor quantum yields (4% and 0.022%) and moderate *g*_lum_ of 0.028 and 0.020 for Eu^3+^ and Tb^3+^, respectively (Muller et al., 2002). Along this line, the same authors used enantiopure dipicolinic acid-based ligands, which produce helical arrangements around the metal center mirroring those previously described for tpy-based complex ligands (Golesorkhi et al., 2018). At this occasion, they observed a considerable improvement of the chiroptical properties by measuring a *g*_lum_ of −0.18 for the Eu^3+^ derivative (Bonsall et al., 2007). Other important works using dipicolinic acid-based ligands have been reported, some of them leading to supramolecular structures (Leonard et al., 2007; Lincheneau et al., 2011; Kitchen et al., 2012; Bradberry et al., 2015; Zhang G. et al., 2015).

**Figure 4:**
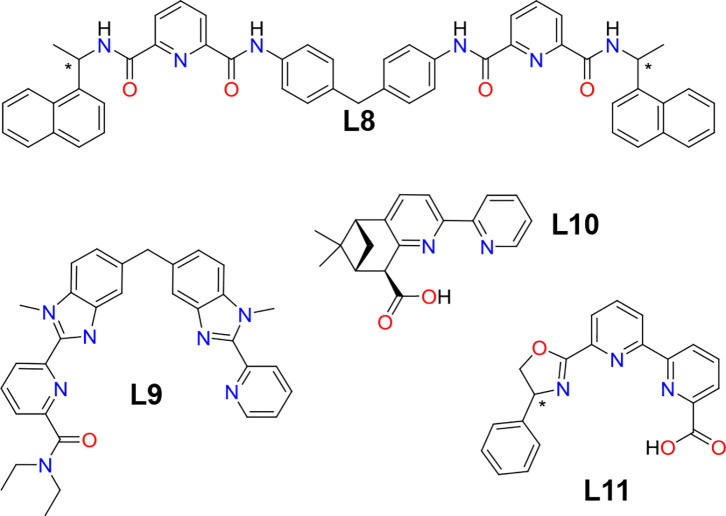
Multidentate ligands L8, L9, L10, and L11 that have been used for the preparation of chiral supramolecular assemblies, such as helicates (L8 and L9), trinuclear assemblies (L10), and wheels (L11) mainly by complexation to Eu^3+^.

So far, we have focused our analysis on small chiral complexes, but more sophisticated structures, such as helicates, cages, or wheels showing interesting CPL properties have also been reported (Figure 4). For instance, Piguet and coworkers prepared the first enantiomerically pure inert nonadentate receptor [Cr(**L9**)_3_]^3+^ (Cantuel et al., 2004). Its complexation to Ln^3+^ generates the triple-stranded helicates [LnCr(**L9**)_3_]^6+^ (e.g., Ln = Eu, Tb), the CPL spectrum of which revealed a dual polarized emission for [EuCr(**L9**)_3_]^6+^ arising from the spin–flip Cr^III^(^2^E → ^4^A_2_) transition with |*g*_lum_| = 0.01 at 744 nm and from the Eu^III^(^5^${\text{D}}_{0}\to^{7}$F_1_) transition with |*g*_lum_| = 0.16 at 594 nm. Due to the quantitative intramolecular Tb → Cr energy transfer, [TbCr(**L9**)_3_]^6+^ showed exclusively the polarized emission of Cr^3+^ with a *g*_lum_ value identical to that observed for the Eu derivative. Mamula et al. were able to prepare a trinuclear Eu_3_(**L10**)_2_ array through a diastereoselective assembly process harnessing the so-called chiral cooperativity approach and using an enantiopure bipyridine-carboxylate ligand (**L10**, |*g*_lum_| = 0.16 for the Eu(^5^${\text{D}}_{0}\to^{7}$F_1_) transition) (Mamula et al., 2005). Similarly, Gunnlaugsson and coworkers prepared the [Eu_2_(**L8**)_3_]^6+^ triple-stranded helicate by self-assembly of enantiomerically pure **L8** and Eu(CF_3_SO_3_)_3_ salts (Stomeo et al., 2009). CPL measurements showed a large *g*_lum_ of −0.23 at 593 nm corresponding to the magnetic allowed ^5^${\text{D}}_{0}\to^{7}$F_1_ transition. Mazzanti and coworkers prepared a chiral heptanuclear Eu^3+^ wheel using the dissymmetric tetradentate ligand **L11** (Bozoklu et al., 2012). This system showed a reasonable quantum yield of 27% with a |*g*_lum_| of 0.1 for the ^5^${\text{D}}_{0}\to^{7}$F_1_ transition. Other assemblies, such as tetrahedral and cubic cages, triangles, wheels, and clusters have been also reported, most of them based on Eu^3+^ with |*g*_lum_| ranging from 0.04 to 0.20 (Mamula et al., 2006; Tang et al., 2009; Yan et al., 2015; Li X.-Z. et al., 2017; Yeung et al., 2017; Bing et al., 2018; Zhou et al., 2019).

Although far to be comprehensive, the selected contributions commented above represent important and illustrative results in the field of lanthanide's CPL. The usual strategy to prepare chiral Ln^3+^-based complexes deals with the introduction of stereogenic centers in the ligand skeleton that itself should (i) act as an efficient antenna for inducing the lanthanide emission and (ii) provide a DC mechanism for inducing considerable CPL. Thus, chiral Eu^3+^, Tb^3+^, and Sm^3+^ complexes have been largely exploited and they are now well-recognized for having strong CPL in the visible range whereas Yb^3+^ and Nd^3+^ centers have also been proven to show remarkable CPL emission in the NIR. This has led to the preparation of a plethora of optically active complexes with reasonable quantum yields. Despite the many examples reported to date, the relationship between the structure of the complex and the value of *g*_lum_, which is of fundamental importance for molecular programming, remains challenging. This weakness mainly relies on the difficulties of performing theoretical CPL studies accessible to synthetic coordination chemists (Gendron et al., 2019). The potential applications of CPL arising from chiral Ln^3+^ systems is of considerable importance in biology and biochemistry because they have shown to be very sensitive to small changes in the molecular structure which can be detected as a change in the CPL emission. Therefore, they can be used as molecular probe for amino acid, anion, and biomolecule sensing. However, the high cost owed to the difficult purification of lanthanides and the theoretical complications associated with the operation of intermediate to strong spin–orbit coupling are non-negligible limitations for applications. Therefore, alternatives regarding the replacement of the optically active lanthanide center with transition metals might be of interest. Furthermore, the chemical lability of most of lanthanide complexes (apart from cryptates or anionic multidentate podates) is a severe drawback that usually prevents chiral resolution.

## Second- and Third-Row Transition Metal Complexes for CPL

**Figure 5:**
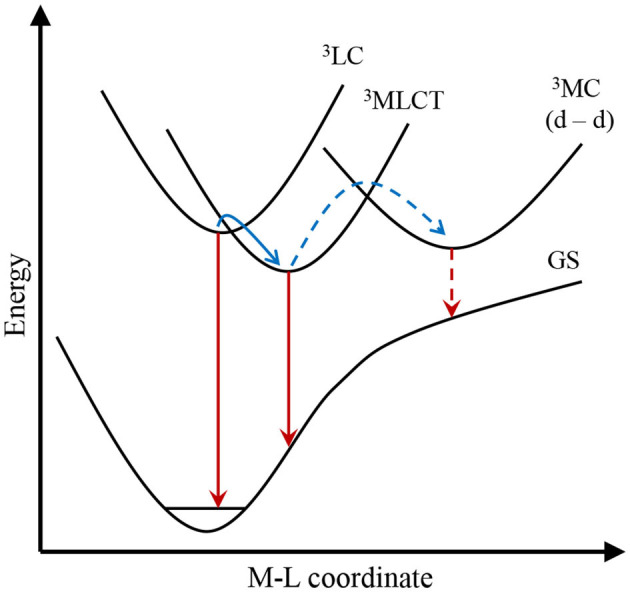
Energy diagram established for a d^6^ second- or third-row transition metal complex showing relaxation processes from triplet states via emission (red arrows) and energy transfer (blue arrows). The manifold ^3^LC/^3^MLCT emission and conversion are highlighted with plain arrows, whereas the potential quenching by the ^3^MC state is shown with dashed arrows.

The 4d and 5d metal complexes benefit from their kinetic inertness, which allows their stereoisomeric separation, purification, and easy incorporation into devices. Those chemical advantages are supplemented by the strong and tunable emission properties covering the visible part of the electromagnetic spectrum with sometimes impressive quantum yields, which are important advantages for modern applications (i.e., OLED or bio-probes) (Guerchais and Fillaut, 2011; Zhou et al., 2011). The efficient emission from the ^3^LC or ^3^MLCT transitions is due to the strong ligand field of complexes holding large metal ions and showing important covalence. In particular, the low spin d^6^ (Ru^II^, Os^II^, Rh^III^, Ir^III^) or d^8^ (Pt^II^) configurations in octahedral and square planar geometries, respectively, promote the ^3^MC quencher levels to high energy, which prevents ^3^LC/^3^MLCT → ^3^MC energy transfers (Figure 5). Increasing the emission quantum yield is thus indebted to the ligand field increase, a concept often harnessed to generate more efficient Ru^II^ emitters.

**Figure 6:**
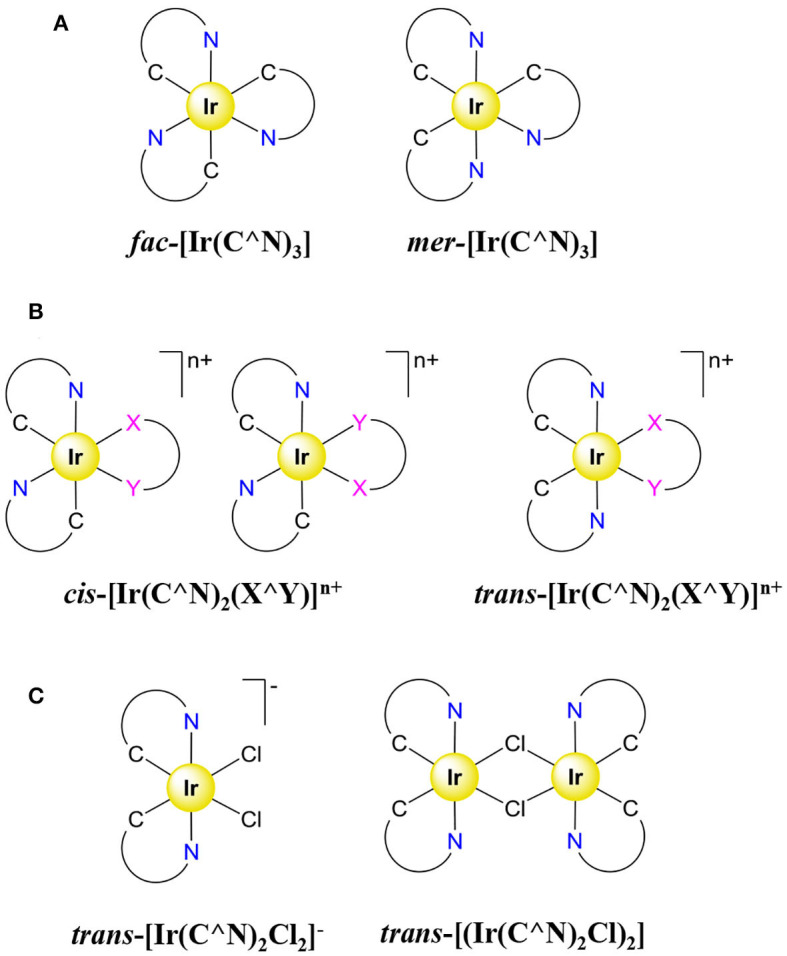
Representation of all the configurational isomers for **(A)** a homoleptic [Ir(C^∧^N)_3_] complex, **(B)** a heteroleptic [Ir(C^∧^N)_2_(X^∧^Y)]^n+^ complex. **(C)** *Trans* precursors [Ir(C^∧^N)_2_Cl_2_]^−^ (left) and [(Ir(C^∧^N)_2_Cl)_2_] (right) for the synthesis of heteroleptic [Ir(C^∧^N)_2_(X^∧^Y)]^n+^ complexes.

Among the d^6^ metals, the strong-field and chiral emissive Ir^III^ cyclometallated complexes cover the visible spectral range and have been thus widely used as CPL emitters (Han et al., 2018). This important feature together with their appealing total luminescence properties have opened new perspectives for life science applications (Caporale and Massi, 2018), bio-imaging (Lo et al., 2011), electroluminescence (Kapturkiewicz, 2016), phototherapy (Zamora et al., 2018), and OLEDs (Zhou et al., 2011). The emission of the Ir^III^ cyclometallated complexes arised from a ^3^LC/^3^MLCT manifold displaying excited states lifetimes around 50 and 5 μs, respectively (Flamigni et al., 2007). Harnessing the Ir^III^ emission for CPL requires chiral complexes, and apart from a sole example of a chiral complex [Ir(N^∧^C^∧^N′)(C^∧^N)X] (X^−^ = Cl^−^ or CN^−^) holding a tridentate, a bidentate, and a monodentate ligand, the enantiomers of which were separated by chiral HPLC (*g*_lum_ = 4 × 10^−3^) (Ashizawa et al., 2009), the chiral environments of Ir^III^ complexes were mainly produced with the help of pseudo-octahedral ter-bidentate environments leading to Δ/Λ stereoisomers in the [Ir(X^∧^X)_3_]^n+^ complexes. Moreover, the use of unsymmetrical cyclometalated ligands in ter-bidentate [Ir(X^∧^Y)_3_]^n+^ complexes induces the potential formation of *fac*/*mer* configurational isomers (Figure 6A); a synthetic issue that could be partially overcome thanks to the so-called *trans* effect since *mer* and *fac* isomers can be quantitatively formed, respectively at low (meridional conformation, kinetic product) and high temperatures (facial isomer, thermodynamic product) (Tamayo et al., 2003). The formation of heteroleptic complexes [Ir(C^∧^N)_2_(X^∧^Y)]^n+^ further increases the number of available isomers (Figure 6B). Owing to the easy access to the intermediates [Ir(C^∧^N)_2_Cl_2_]^−^ or [(Ir(C^∧^N)_2_Cl)_2_] showing N–Ir–N *trans* configuration (Figure 6C), all heteroleptic Ir^III^ complexes of [Ir(C^∧^N)_2_(X^∧^Y)]^n+^ type reported to date for CPL investigations have been designed to possess a N–Ir–N *trans* configuration in order to avoid mixture of isomers.

**Figure 7:**
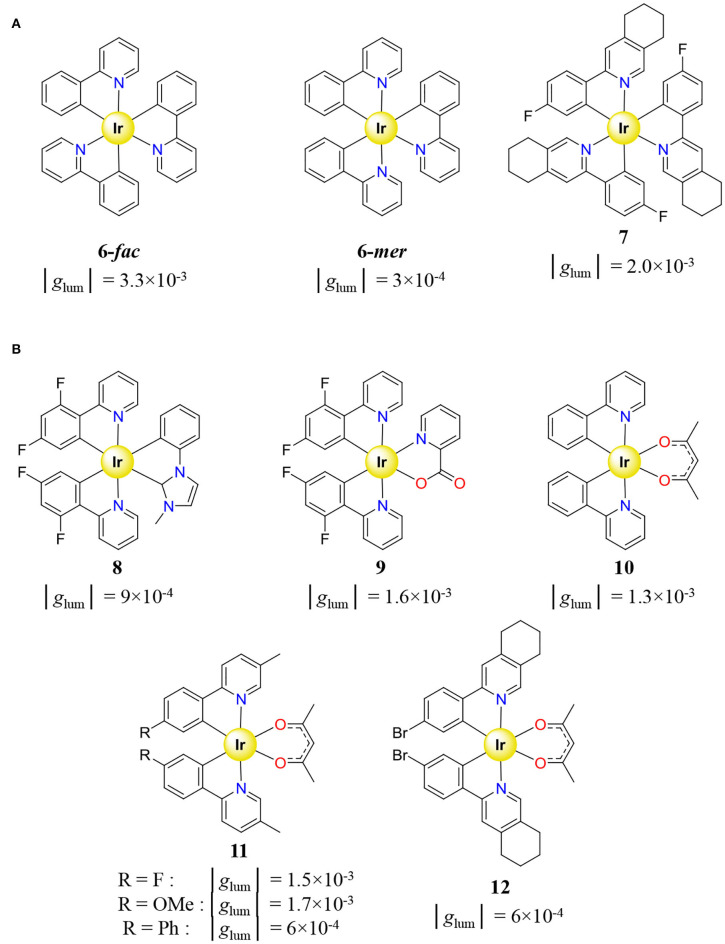
**(A)** Homoleptic [Ir(C^∧^N)_3_] (Coughlin et al., 2008; Li et al., 2015) and **(B)** heteroleptic [Ir(C^∧^N)_2_(X^∧^Y)] (Coughlin et al., 2008; Li et al., 2015; Hellou et al., 2017), cyclometallated Ir^III^ complexes holding achiral ligands displaying CPL, and associated dissymmetry factor *g*_lum_.

In order to obtain enantiopure ter-bidentate Ir^III^ complexes for CPL, a first strategy relies on a unique source of chirality induced by the metal taken as the stereogenic center and using achiral bidentate ligands. Such method produces a pair of helical enantiomers as a racemate which should be further resolved by chiral HPLC. The latter separation technique was successfully achieved for simple mixtures of *fac*/*mer*-[Ir(ppy)_3_] (**6**) (ppy = 2-phenylpyridine) complexes to give *mer*-Λ-[Ir(ppy)_3_], *mer*-Δ-[Ir(ppy)_3_], *fac*-Λ-[Ir(ppy)_3_], and *fac*-Δ-[Ir(ppy)_3_] (Li et al., 2015), as well as for the more sophisticated complex **7** (Figure 7A) (Coughlin et al., 2008). Interestingly, the dissymmetry factor of *fac*-**6** is one order of magnitude superior to its *mer* counterpart, which might explain the popularity of *fac* isomers in CPL.

**Figure 8:**
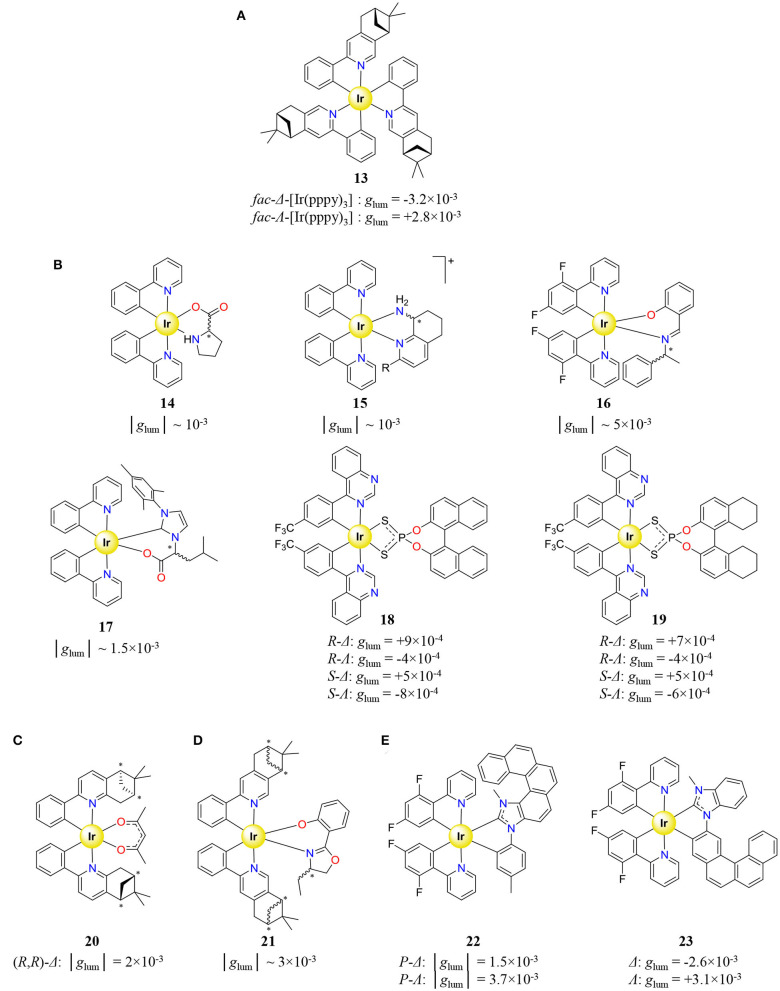
Cyclometallated Ir^III^ complexes holding chiral ligands, displaying CPL, and associated dissymmetry factor *g*_lum_. **(A)** Homoleptic *fac*-[Ir(pppy)_3_] complex (Schaffner-Hamann et al., 2004). Heteroleptic complexes [Ir(C^∧^N)_2_(X^∧^Y)]^n+^ holding **(B)** achiral C^∧^N and chiral X^∧^Y ligands (Li et al., 2016; Mazzeo et al., 2016; Li L.-P. et al., 2017; Manguin et al., 2019; Yan et al., 2019a), **(C)** chiral C^∧^N and achiral X^∧^Y ligands (Yang et al., 2009), **(D)** chiral C^∧^N and X^∧^Y ligands (Yan et al., 2018), and **(E)** helicenic–NHC chiral ligand X^∧^Y (Hellou et al., 2017; Macé et al., 2019).

The previously introduced synthetic method for preparing [Ir(C^∧^N)_2_(X^∧^Y)]^n+^ afforded some heteroleptic complexes holding different achiral ligands (Figure 7B). Because of the use of unresolved [Ir(C^∧^N)_2_Cl_2_]^−^ or [(Ir(C^∧^N)_2_Cl)_2_] precursors, chiral resolution of the synthetized Δ/Λ-[Ir(C^∧^N)_2_(X^∧^Y)]^n+^ racemate **8** (Hellou et al., 2017), **9**, **10** (Li et al., 2015), and **11**, **12** (Coughlin et al., 2008) was performed by chiral supercritical fluid chromatography, before CPL measurement. Since the isolation of pure enantiomers requires sophisticated chiral resolution, the latter method is neither the most popular nor the first exploited to generate enantiopure Ir^III^ complex for CPL. An alternative strategy exploits the coordination of an enantiopure chiral ligand to Ir^III^, which provides a pair of diastereoisomers allowing (i) chiral induction, (ii) quantification of diastereomeric excess by NMR, and (iii) diastereoisomer separation by conventional chemical techniques, such as crystallization, precipitation, or chromatography. Actually, the first CPL measurement performed on an Ir^III^ complex used an homoleptic [Ir(pppy)_3_] (**12**) complex (pppy = (8R,10R)-2-(2′-phenyl)-4,5-pinenopyridine) synthetized according to the chiral induction methodology (Figure 8A) (Schaffner-Hamann et al., 2004). The reaction of Ir(acac)_3_ with pppy at 190°C in glycerol afforded pure *fac*-[Ir(pppy)_3_] with a diastereomeric Λ/Δ ratio of 2/3. Further separation of the two diastereoisomers was achieved on a silica plate and afforded *g*_lum_ of −3.2 × 10^−3^ and +2.8 × 10^−3^ for *fac*-Δ-[Ir(pppy)_3_] and *fac*-Λ-[Ir(pppy)_3_], respectively.

Apart from **13**, the chemical attention usually focused on heteroleptic [Ir(C^∧^N)_2_(X^∧^Y)]^n+^ complexes probably because they are obtained by the simple reaction of the aforementioned Ir^III^ intermediates ([Ir(C^∧^N)_2_Cl_2_]^−^ or [(Ir(C^∧^N)_2_Cl)_2_]) with enantiopure ligands, a versatile strategy which produces a library of complexes with various chiral ligands. In particular, the combination of achiral C^∧^N ligands with a chiral X^∧^Y one afforded the most important library of [Ir(C^∧^N)_2_(X^∧^Y)]^n+^ complexes for CPL, mainly based on central chirality (**14**–**17**) (Li et al., 2016; Mazzeo et al., 2016; Li L.-P. et al., 2017; Manguin et al., 2019), albeit axial chirality was occasionally exploited with ligands containing 1,1′-bi-2-naphthol derivatives (**18**–**19**) (Figure 8B) (Han et al., 2017b; Yan et al., 2019a). Although a few examples report on full chiral induction (**14**) (Li L.-P. et al., 2017), in most cases the separation of the formed diastereoisomer pair was achieved by selective diasteroisomer precipitation (**15**) (Mazzeo et al., 2016), by HPLC (**16**) (Li et al., 2016), or by silica column chromatography (**17**–**19**) (Manguin et al., 2019; Yan et al., 2019a). Alternatively, the ligand chirality can be introduced on the C^∧^N ligand held by the precursor Ir^III^ complex prepared as an unresolved diasteroisomeric mixture. Hence, the coordination of acac (X^∧^Y) to a precursor containing enantiopure 2-phenyl-5,6-pinenepyridine (C^∧^N) resulted in a surprising full chiral induction with the exclusive formation of the Δ compound (**20**) (Figure 8C) (Yang et al., 2009). Finally, the introduction of both enantiopure C^∧^N and X^∧^Y ligands provided 8 stereoisomers from the overall 3 stereogenic centers (**21**) (Figure 8D) (Yan et al., 2018). The various mixtures of diastereoisomers could be successfully separated by silica column chromatography. Even if chiral induction is an outstanding strategy for the easy preparation of stereoisomerically pure Ir^III^ complexes, this method requires kinetically inert enantiopure ligands. Nevertheless, the synthesis of a [Ir(C^∧^N)_2_(X^∧^Y)]^*n*+^-type complex with a helicenic–NHC as ligand X^∧^Y (**22**–**23**) was conducted despite the observed racemization of the unbound helicene (Figure 8E) (Hellou et al., 2017; Macé et al., 2019). Since racemization is inhibited upon coordination, the chiral resolution of the four stereoisomers could be performed by chiral HPLC. In spite of all the remarkable synthetic efforts made for generating libraries of enantiopure Ir^III^ emissive complexes, the CPL measurements always show weak and comparable dissymmetry factors in the range of 10^−4^ -5 × 10^−3^, an observation which is consistent with the nature of the electric-dipole allowed transitions characterizing mixed ^3^LC/^3^MLCT manifolds.

**Figure 9:**
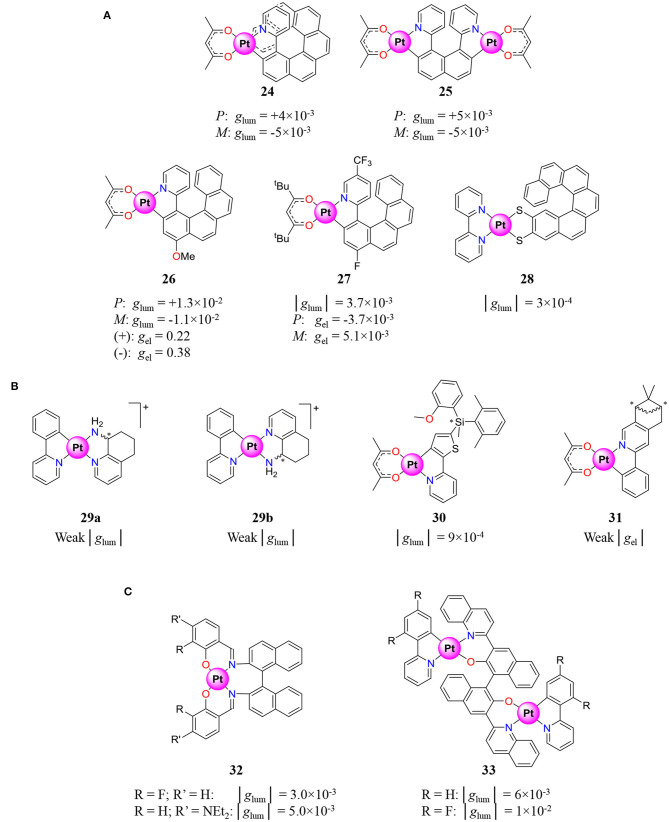
Pt^II^ complexes coordinated by **(A)** helicene ligands (Shen et al., 2014; Brandt et al., 2016; Biet et al., 2017; Yan et al., 2019b), **(B)** bidentate ligands showing central chirality (Ionescu et al., 2017; Lu et al., 2017; Usuki et al., 2019), and **(C)** bidentate ligands showing axial chirality (Song J. et al., 2018; Song et al., 2019), and associated luminescence dissymmetry factor *g*_lum_ and electroluminescence dissymmetry factor *g*_el_.

Recently, neutral Pt^II^ complexes received increasing attention as CPL emitters because they combine (i) chemical and kinetic advantages similar to Ir^III^ complexes, (ii) important emission quantum yield from ^3^MLCT state or even ^3^MMLCT state due to Pt–Pt bonds, and (iii) emission in the red-to-NIR region, which is an asset for biological applications. Pt^II^ (d^8^) complexes usually display square planar geometry with a coordination number of 4 compatible with additional intermolecular axial interactions produced by molecule pilling which permit the tuning of the ^3^MMLCT emission energy via Pt–Pt bond formation. Contrary to aforementioned pseudo-octahedral Ir^III^ ter-bidentate complexes, the square planar Pt^II^ ones possess an intrinsically achiral first coordination sphere, which affords an easy route for the preparation of enantiopure complexes. Indeed, the coordination of chiral enantiopure ligands generates the corresponding complex without the appearance of an additional stereogenic center upon coordination—a convenient synthetic pathway, which makes Pt^II^ complexes particularly appealing for CPL. The first CPL of a Pt^II^ complexes was thus reported by Crassous and coworkers in 2014. They correspond to the coordination of cyclometalated bidentate helicene ligands, with an acac anion completing the coordination sphere (**24**–**26**) (Figure 9A) (Shen et al., 2014). While **24** and **25** display weak dissymmetry factors, the related complex **26** shows *g*_lum_ one order of magnitude higher than that measured for the Ir^III^ analogs, this despite the simple ligand-centered chirality. This confirms the efficiency of the DC mechanism and the option of avoiding metal-centered chirality for producing large CPL signals. Owing to its outstanding *g*_lum_ = 1.3 × 10^−2^ for a d-block complex, **26** was recently incorporated into an electroluminescent device which provided an electroluminescent dissymmetry factor (*g*_el_) one order of magnitude higher than its photoluminescent counterpart (*g*_lum_)—a result probably due to an additional helical arrangement in the condensed phase (Brandt et al., 2016). The impressive *g*_el_ is sensitive to weak structural modifications since the similar complex **27** showed much weaker values (Yan et al., 2019b). Motivated by the seminal work of Crassous and coworkers, numerous chiral Pt^II^ complexes for CPL studies were synthesized with the help of bidentate ligands possessing helical chirality (**27**–**28**, Figure 9A) (Biet et al., 2017), central chirality (**29**–**31**, Figure 9B) (Ionescu et al., 2017; Lu et al., 2017; Usuki et al., 2019), or axial chirality (**32**–**33**, Figure 9C) (Song J. et al., 2018; Song et al., 2019).

**Figure 10:**
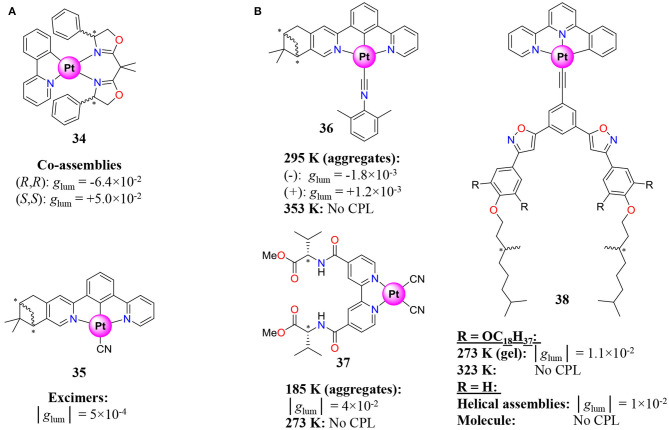
Pt^II^ complexes **(A)** showing aggregation (Tanaka et al., 2016; Park et al., 2019), **(B)** showing reversible aggregation and switchable CPL and associated dissymmetry factor *g*_lum_ (Ikeda et al., 2015, 2018; Zhang X.-P. et al., 2015; Fu et al., 2016).

The fluorinated version of the bimetallic complex **33** shows a *g*_lum_ of 10^−2^ due to the helical shape induced by the BINOL ligand, which highlights that the pilling of complexes in an overall helical architecture is a promising strategy for *g*_lum_ enhancement (Song et al., 2019). The latter strategy was repeated with complex **34**, which is used as a dopant in achiral analog for the formation of helical co-assemblies with improved *g*_lum_ for the ^3^MMLCT transition via Pt–Pt bonding interactions (Figure 10A) (Park et al., 2019).

**Figure 11:**
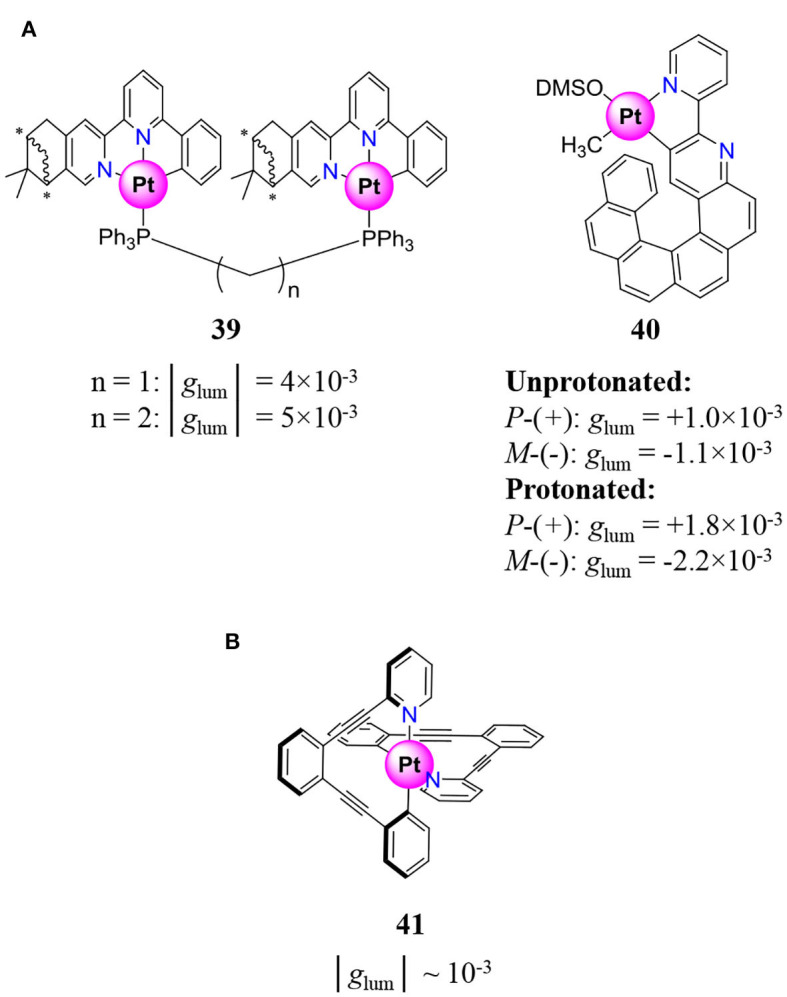
**(A)** Pt^II^ complexes showing CPL signals tunable by spacer length (left) or switchable by protonation/deprotonation (right) (Saleh et al., 2015a; Zhang et al., 2018). **(B)** Pt^II^ cyclometallated complexes holding achiral ligands and showing metal centered chirality (Schulte et al., 2017), and associated dissymmetry factor *g*_lum_.

In order to provide efficient stacking and intermolecular Pt–Pt bonding interactions, Pt^II^ complexes holding a tridentate and a monodentate ligand were developed (**35**, **36**, **38**, Figure 10) (Ikeda et al., 2015, 2018; Zhang X.-P. et al., 2015; Tanaka et al., 2016). Despite positioning the stereogenic centers on the ligand far from the emissive moiety, the resulting aggregates and helical supramolecular structures often display important dissymmetry factor around 10^−2^. Moreover, the reversible entropic control of the aggregation process transforming CPL active assemblies (at low *T*) into discrete molecules with no CPL signal (at high *T*) provides a noteworthy CPL switch (**36**–**38**, Figure 10B) (Ikeda et al., 2015, 2018; Zhang X.-P. et al., 2015; Fu et al., 2016). The tuning of the CPL property can nonetheless be achieved in solution by controlling the intermetallic distance in a bimetallic architecture *via* the choice of the spacer length between complexes (**39**, Figure 11A) (Zhang et al., 2018).

A remarkable modulation of the CPL property in solution could be achieved with the cyclometallated Pt^II^-helicene complex (**40**, Figure 11A), for which reversible protonation of the bidentate helical ligand provided a modification of *g*_lum_ albeit its absolute value remains weak (Saleh et al., 2015a). Aside from complexes owing their chirality to stereogenic centers located on the ligand, Clever and coworkers reported in 2017 a remarkable Pt^II^ cyclometallated complex built with an achiral ligand and showing metal-centered chirality despite the square planar geometry of the first coordination sphere (**41**, Figure 11B) (Schulte et al., 2017). In this complex, the chirality arises from an appropriate choice of the ligand coordination vectors, which permit the unusual orthogonal coordination of the two bidentate ligands.

**Figure 12:**
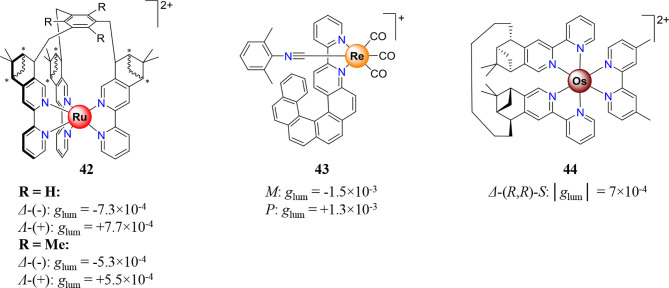
Ru^II^ **(left)** (Oyler et al., 2007), Re^I^ **(center)** (Saleh et al., 2015b), and Os^II^ **(right)** (Gunde et al., 1997) complexes showing CPL, and associated dissymmetry factor *g*_lum_.

Beyond the most popular Ir^III^ and Pt^II^ neutral complexes, the CPL of second- and third-row transition metal complexes is currently restricted, to the best of our knowledge, to three reports dealing with chiral and charged Ru^II^ (**42**) (Oyler et al., 2007), Re^I^ (**43**) (Saleh et al., 2015b), and Os^II^ (**44**) (Gunde et al., 1997) complexes (Figure 12).

The use of [Ru^II^(R-bipy)_3_] chromophores restores the difficulties associated with the prospective formation of Δ/Λ stereoisomers as well as *fac*/*mer* regioisomers. Nevertheless, those synthetic drawbacks were finally overcome by the use of enantiopure ter-bidentate podand, the coordination of which solely provides the *fac* isomer **42** (Oyler et al., 2007). Moreover, the three stereogenic centers located on the hexadentate ligand induce the complete diastereoselective formation of the Δ-(-)-**42** and Λ-(+)-**42** complexes which showed weak *g*_lum_ value of 7.7 × 10^−4^. More recently, the complex **43** was obtained through the coordination of a helicenic ligand to a Re^I^ metal center with intrinsic achiral coordination sphere (Saleh et al., 2015b). Although this straightforward method provides enantiopure complexes, the associated dissymmetry factors remain weak, and only few further synthesis were performed for CPL application (Gauthier et al., 2020). Finally ligand-centered CPL were casually tuned or switched by a closed shell d-block metal like Ag^I^ or Au^I^. While Au^I^ can be used to rigidify and organize organic chromophores to enhance its emission (Zhang J. et al., 2019), the interaction of Ag^I^ with organic moieties provides CPL switch and ratiometric CPL probe (Reiné et al., 2018a; Resa et al., 2018). Furthermore, CPL emission can be observed from nanoclusters of silver or gold in their metallic state (Kumar et al., 2017; Shi et al., 2017).

Excluding the outstanding electroluminescence of **26**, the CPL-active 4d and 5d metal complexes reported to date display a systematically weak dissymmetry factor (<7 × 10^−2^) in line with the limitations ascribed to ED-allowed and MD-forbidden charge transfer or ligand-centered transitions. Similarly to the lanthanide-based f–f transitions, the d–d transitions in the second and third-row metal complexes should display strong CPL, albeit there are non-emissive because of the low-lying MLCT. In order to observe emissive metal centered d–d transitions, the strong ligand field of 4d and 5d metal complexes should be reduced until the energy of MC states becomes lower than those of MLCT states, a criterion more easily fulfilled with 3d metal complexes.

## Extension Toward 3D Metal Complexes. The Promising Case of Earth-Abundant Chromium Centers

The earth-abundant first-row d-block metals seem appealing in the field of CPL because of their low cost and the theoretically promising dissymmetry factors arising from the d–d transitions. Assuming very weak spin–orbit coupling and pure metal character of d orbitals, the d–d transitions with Δ*L* = 0 are indeed ED-forbidden and MD-allowed. However, pure metal-centered emission in 3d coordination complexes, as found in 4f-block analogs, is rare because of the considerable covalencies of the M–L bonds that promote mixing with ligand-based wave functions, efficient coupling with vibration modes, and some significant quenching of the luminescence. Regarding this last point, a favorable situation for an emissive 3d metal excited level requires (i) a minimum perturbation by the ligand field in order to retain the almost pure d–d character of the transition and (ii) an important energy gap with upper excited levels in order to prevent deleterious back inter system crossing (BISC). Because of those sophisticated conditions, the use of first-row metals is mainly restricted to closed shell ions with d^10^ configuration, such as Zn^II^ complexes showing ligand-based emission (Isla et al., 2016; Kögel et al., 2016; Aoki et al., 2017; Reiné et al., 2018b; Maeda et al., 2019), or in Cu^I^ complexes displaying MLCT emission (Zhang M.-M. et al., 2019; Deng et al., 2020). In the first case, the Zn^II^ ions are responsible, upon their coordination to an organic ligand, for conformational changes affecting the optical and chiroptical properties, particularly in the CPL emission. However, *g*_lum_ remains low ranging from 10^−2^ to 10^−4^ as it arises from the organic moiety of the complex. One can note that chiral porphyrin-based ligand platforms have been widely used for such purposes. Interestingly, Cu^I^ has been recently exploited for the preparation of enantiopure organometallic complexes based on chiral carbene ligands. These complexes have the advantage of being prepared directly from enantiopure ligands and so there is no need for chiral resolution. However, the highest *g*_lum_ value recorded up to now reaches only 10^−3^, but this strategy opens a new way to prepare enantiopure complexes based on earth-abundant metals acting as CPL emitters.

**Figure 13:**
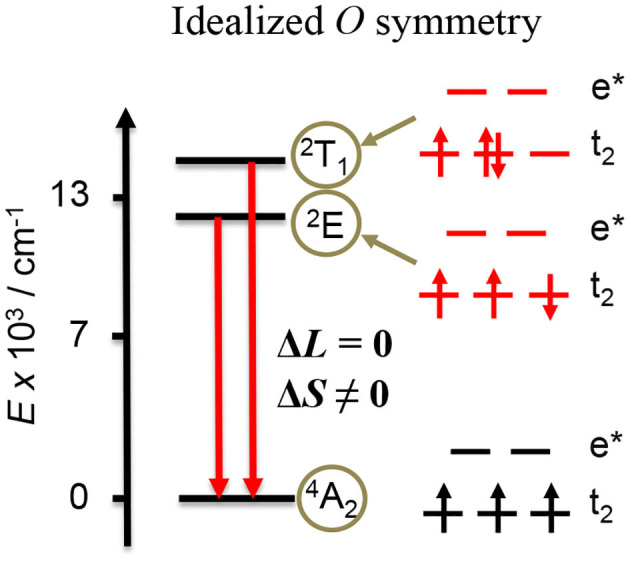
Low-energy part of the energy level diagram for a Cr^III^N_6_ chromophore in octahedral symmetry. The two red arrows represent the emissive spin–flip transitions from the two low-lying excited states (^2^T_1_ and ^2^E). The electronic arrangement is highlighted in the right part of the diagram.

Significant *g*_lum_ enhancement thus requires moving from charge transfer to ED-forbidden/MD-allowed metal-centered transitions operating in 3d open-shell metal ions, but only few studies were reported on first-row transition metals, mainly on Cr^III^ and once on Mn^II^ showing CPL in the solid state (Zhao et al., 2019). Although the use of labile Mn^II^ is compatible with chiral induction produced by enantiopure ligands, the ideal metal complexes should be inert enough to tolerate separation techniques, such as chromatogaphy, a criterion only fulfilled with Co^III^, Fe^III^, or Cr^III^. While the two former ions are known to be non-emissive, Cr^III^ complexes are known to show NIR emission in the 600–800 nm region, arising from the two low-lying Cr^III^(^2^E) and Cr^III^(^2^T_1_) spin–flip excited states (Figure 13) (Jamieson et al., 1981; Kirk, 1981, 1999; Forster, 1990, 2002; Buldt and Wenger, 2017; Otto et al., 2018). In fact, Cr^III^ complexes have long been known to exhibit interesting photophysical and photochemical properties as illustrated by the archetypal ter-bidentate [Cr(phen)_3_]^3+^ (Serpone et al., 1979; Ryu and Endicott, 1988; Isaacs et al., 2006; Donnay et al., 2007; Vandiver et al., 2010; Vasudevan et al., 2010; Doistau et al., 2018, 2020), bis-terdentate [Cr(tpy)_2_]^3+^ (Serpone et al., 1979; Scarborough et al., 2012; Constable et al., 2014; Schönle et al., 2015a,b; Barbour et al., 2017), and analogs (Zare et al., 2017b; Jiménez et al., 2018). In all cases, the ligand-field strength induced by the phen and tpy ligands is strong enough to induce near-infrared phosphorescence arising from the lowest-lying Cr(^2^T_1_) and Cr(^2^E) excited states, but the associated very low quantum yields (<0.2%) are strong limitations for their use as CPL probes. Thus, much effort was focused on the optimization of the Cr^III^ complexes in order to improve their photophysical properties. The introduction of six-membered chelated rings in the terdetante ligands (ddpd = *N, N*′-dimethyl-*N, N*′-dipyridin-2-ylpyridine-2,6-diamine or dqp = 2,6-di(quinolin-8-yl) ligands) has proven to be an efficient approach to increase both the quantum yield and the lifetimes of the Cr(^2^T_1_) and Cr(^2^E) excited states in [Cr(ddpd)_2_]^3+^ and [Cr(dqp)_2_]^3+^ (Otto et al., 2015; Wang et al., 2017; Jiménez et al., 2018, 2019; Treiling et al., 2019).

In the frame of CPL, Cr^III^ complexes holding strong nitrogen donors as ligands are valuable candidates because the energy gap with the upper ligand field state (^4^T_2_) is large enough to prevent BISC and subsequent quenching (Fucaloro et al., 1983; Doistau et al., 2018; Otto et al., 2018). In addition, the Tanabe–Sugano diagram pertinent to a d^3^ electronic configuration indicates that the Cr^III^(^2^T_1_,^2^E) energy levels are almost insensitive to the ligand field strength, which preserve the ED-forbidden/MD-allowed character of the Cr^III^(^2^T_1_,^2^E → ^4^A_2_) transitions (Lever, 1984; Zare et al., 2017b; Jiménez et al., 2018). Hence, the Cr^III^N_6_ complex NIR emission seems to fulfill the established criteria for exhibiting strong CPL with high *g*_lum_. It is however worth stressing here that, although the spin–flip Cr^III^(^2^T_1_,^2^E → ^4^A_2_) emissive transitions are both strictly Laporte and spin-forbidden, the former rule is released in non-centrosymmetric systems and considerable intensity can be also gained through the vibronic coupling with unsymmetrical modes. Hence, the distortion from a perfect centrosymmetric octahedral should have a deleterious effect on the ED/MD ratio because of increasing covalence in the t_2g_ orbitals—a consequence also triggered by strong π interactions and by strong nephelauxetic effect. Finally, the Cr^III^N_6_ complexes may allow discriminating the contribution of both ligand-field splitting and Racah parameters to the magnitude of *g*_lum_–a feature which remains unexplored. During the early seventies, there was an ephemeral interest for chiral Cr^3+^ complexes that gave rise to the first reports on the circularly polarized emission arising from first raw transition metal complexes. In their seminal work, Emeis and Oosterhoff reported in 1967 that the simple chiral complexes [Cr(en)_3_]^3+^ (en = ethylenediamine) showed, despite a weak quantum yield, a remarkable |*g*_lum_| = 0.028 for the spin–flip Cr^III^(^2^E → ^4^A_2_) transition (Emeis and Oosterhoff, 1967), a result boosted to 0.046 in an ethylene glycol/water solution by Richardson and coworkers 10 years later (Hilmes et al., 1977). This |*g*_lum_| value is quite high compared with p-block systems and with 4d- and 5d-block-based complexes, which confirms the potential of Cr^III^ ions for inducing large *g*_lum_. Despite those promising results, only scarce studies focused on Cr^III^ complexes reported CPL measurements in condensed phases (Manson and Shah, 1977; Yamaga et al., 1990; Nobuhiro et al., 2001; Anzai et al., 2003, 2006; Seyler et al., 2018), or in solution except for some triple helical complexes [Cr(en)_3_]^3+^ (Madaras and Brittain, 1980; Morita et al., 1984; Herren et al., 1996), [Cr(ox)_3_]^3+^ (ox = oxalate) (Herren et al., 1996), [Cr(pn)_3_]^3+^ (pn = 1,2-propanediamine) (Morita et al., 1984; Herren et al., 1996), and [Cr(ala)_3_] (ala = alaninato) (Tsubomura et al., 1988; Taro et al., 1991).

**Figure 14:**
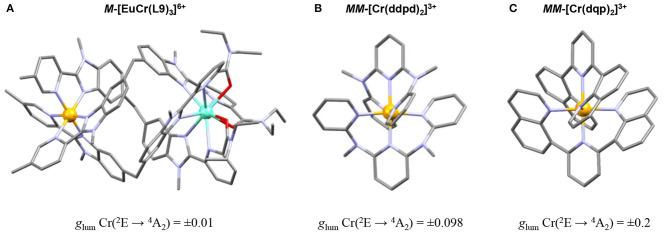
**(A–C)** Crystal structures of chiral Cr^III^ based polypyridyl complexes and their respective *g*_lum_ values for the spin–flip Cr^III^(^2^E → ^4^A_2_) transition. Color code: Cr (orange), Eu (sky blue), C (gray), N (purple), O (red), H, and counter-ions are omitted for clarity.

In 2001, Piguet and coworkers took advantage of the Cr^III^ complex inertness to prepare and resolve the enantiomerically pure triple-stranded helicates [LnCr(**L9**)_3_]^6+^ (e.g., Ln = Eu, Tb) (Figure 14A) which revealed Cr^III^-centered polarized emission arising from the spin–flip Cr^III^(^2^E → ^4^A_2_) transition with a |*g*_l__um_| of 0.01 at 744 nm (Cantuel et al., 2004). More recently, the chiral resolution of the cationic kinetically inert [Cr(dqp)_2_]^3+^ complex (dqp = 2,6-di(quinolin-8-yl)) displaying extraordinary dual CPL emission was achieved. The wrapped arrangement of the dqp ligand around the Cr^III^ provides a helical configuration which generates a chiral bis-terdentate monometallic Cr^III^ helix as a racemic mixture. The two enantiomers *PP*-[Cr(dqp)_2_]^3+^ and *MM*-[Cr(dqp)_2_]^3+^ (Figure 14C) were separated by chromatography and isolated by cation exchange. The CPL spectra recorded in CH_3_CN solutions showed two polarized emission bands at 724 nm and 747 nm corresponding to the spin–flip Cr^III^(^2^${\text{T}}_{1}\to^{4}$A_2_) and Cr^III^(^2^E → ^4^A_2_) transitions with *g*_lum_ values of ±0.1 and ±0.2, respectively (Jiménez et al., 2019). These values are the highest reported up to date for any transition metal complex, and they are comparable to those found in chiral lanthanide-based f–f complexes. Subsequently, Heinze and coworkers were able to partially resolve the chiral complex [Cr(ddpd)_2_]^3+^ by HPLC (Figure 14B). CPL measurements displayed *g*_lum_ values as high as 0.098 for the Cr^III^(^2^E → ^4^A_2_) transition (Dee et al., 2019).

Interestingly, the [EuCr(**L9**)_3_]^6+^ and [Cr(en)_3_]^3+^ complexes, which show intrinsic chirality of the CrN_6_ coordination sphere due to *D*_3_ local symmetry, display *g*_lum_ significantly lower than the [Cr(dqp)_2_]^3+^ and [Cr(ddpd)_2_]^3+^ complexes where their chirality arises from the helical disposition of the coordination ligands and not from the metal environment. The latter feature highlights the efficiency of the DC mechanism compared to SC. Nevertheless, the distortion from perfect octahedral can be also incriminated since it reduces the pure metal character of the t_2g_ orbitals in the *D*_3_ symmetric coordination sphere holding five-membered chelate rings, while the [Cr(dqp)_2_]^3+^ and [Cr(ddpd)_2_]^3+^ complexes show a nearly undistorted octahedral first-coordination sphere (Perkovic et al., 1991). Finally, the importance for strong CPL due to a helical wrapping of the ligands around the metal should not be underestimated.

In summary, binding semirigid achiral bis six-membered chelate rings (dqp or ddpd) to form helical Cr^III^ complexes and further chiral resolution to generate enantiopure samples makes these systems promising candidates for working as NIR CPL emitters. In these conditions, this earth-abundant metal could become a good competitor to rare earths for chiroptical applications.

## Conclusion

Since the beginning of the twenty-first century, increasing efforts have been devoted to the synthesis of molecules or materials displaying strong CPL with large *g*_lum_. These materials were found appealing for the design of devices, such as CP-OLEDs, the brightness of which can be enhanced compared with their linearly polarized counterparts, or for bio-imaging because of the lower scattering of the circularly polarized light. In terms of processability and adaptability to specific applications, the kinetically inert organic chromophores, as well as 4d and 5d coordination complexes, were systematically exploited despite the rather weak dissymmetry factors (*g*_lum_ <7 × 10^−2^) associated with electric dipole allowed π → π^*^ and charge transfer transitions. Chiral single-wall carbon nano-ring and Pt^II^-helicene complexes have been shown to exhibit the largest *g*_lum_ and *g*_el_ within that family of CPL materials. Improving *g*_lum_ requires ED-forbidden/MD-allowed transition; thus, pure metal-centered transitions (Δ*L* = 0) are probably the best choice. In this context, the f–f transitions benefit from low covalence due to the inner character of the f orbitals, which are responsible for weak crystal field splittings (100–1,000 cm^−1^) and almost pure intrashell character for the f–f transitions. Because of the strong spin–orbit coupling, all f–f transitions are not equally promising for displaying large *g*_lum_ and the target MD-allowed transitions obey specific selection rules applied on Δ*J* values. A large number of chiral lanthanide complexes showing important dissymmetric factors in the range 0.01–2 have been reported with a record value of 1.38 at 595 nm for a europium complex. Nevertheless, the costly lanthanides metals and the kinetic lability of most of Ln^III^-based complexes are severe drawbacks for the preparation of inert enantiopure complexes and their subsequent applications in material science. In order to combine large *g*_lum_, kinetic inertness, and low cost, some 3d metal complexes represent a valuable alternative since the d–d transitions should display similar advantages as those described for f–f, except that they suffer from more important covalence and vibronic coupling. In particular, some Cr^III^ inert complexes have been shown to be compatible with enantiomeric resolution and strong CPL emission due to the spin–flip Cr^III^(^2^T_1_,^2^E → ^4^A_2_) transitions. The pure d–d character of the latter transitions is respected as long as the Cr^III^ coordination sphere is close to a perfect octahedron (no distortion)— a geometry observed for the [Cr(dqp)_2_]^3+^ complex which possesses the record *g*_lum_ value of 0.2 within the family of transition metal complexes. Although a deep theoretical understanding of the *g*_lum_/structure relationship remains somehow elusive, this review dealing with CPL emitters allows the identification of empirical strategies for inducing large *g*_lum_. Firstly, the SC mechanism induced by the intrinsic chirality of the chromophore does not provide the strongest CPL signals. On the contrary, introducing chirality on an aside moiety provides efficient DC mechanism, which has been proven to be an efficient tool for inducing important *g*_lum_–an effect often enhanced when the number of stereogenic moieties increases. Secondly, the favorable MD transition can be enhanced upon implementing (i) helicity *via* aggregate formation or helical ligand coordination/wrapping or (ii) circular skeleton permitting current loop. Finally, in chiral coordination complexes, it is important to reduce the covalence and the mixing of the metal/ligand wave functions in order to retain the Laporte rule (Δ*L* = 0) which controls the intensity of ED and MD transitions. This latter feature is fulfilled for (i) f–f transitions in weak crystal field complexes and for (ii) d–d transitions operating on in close-to perfect centrosymmetrical complexes.

## Author Contributions

BD and J-RJ equally contributed to both bibliography and writing of the manuscript. CP got the financial support for the project, supervised the research group, and corrected the manuscript. All authors contributed to the article and approved the submitted version.

## Conflict of Interest

The authors declare that the research was conducted in the absence of any commercial or financial relationships that could be construed as a potential conflict of interest.
